# The Adolescent Problem Gambling Prevalence Associated with Leisure-Time Activities and Risky Behaviors in Southern Spain

**DOI:** 10.1007/s11469-022-00950-7

**Published:** 2022-11-18

**Authors:** M. Moñino-García, M. Ballesta, JM. Huerta, JF. Correa-Rodríguez, N. Cabrera-Castro, N. Llorens, MD. Chirlaque-López

**Affiliations:** 1 Department of Epidemiology, Regional Health Council of Murcia, Ronda de Levante 11, 30008 Murcia, Spain; 2 Division of Preventive Medicine and Public Health, Department of Public Health Sciences, School of Medicine, University of Murcia, 30100 Espinardo, Murcia, Spain; 3 Institute for Biomedical Research of Murcia, IMIB-Arrixaca, 30120 El Palmar, Murcia, Spain; 4 CIBER Epidemiology and Public Health (CIBERESP), 28029 Madrid, Spain; 5 Spanish Observatory On Drugs, Government Delegation for the National Plan On Drugs, Ministry of Health, Plaza de España, 17, 28008 Madrid, Spain; 6 Faculty of Health Sciences, Valencian International University, C/Pintor Sorolla, 21, 46002 Valencia, Spain

**Keywords:** Problem gambling (Lie-Bet Scale), Preventive factors, Leisure time, Risky behaviors, Compulsive internet use (CIUS scale), Adolescence

## Abstract

Gambling addiction is increasing and is becoming a public health concern due to the rise of gambling-related harms affecting the youth. Previous studies suggest a strong link between problem gambling (PG) and substance use and psychosocial and familial factors. Our main objective was to analyze the association between PG and factors like sport, leisure-time activities, and risk-taking behaviors in adolescents. A survey on substance use and addictive behaviors was performed in 2019 on a representative sample of 2240 subjects (14–18 years) from the Southern Spain Region. Data variables like socio-demographic characteristics, sport, leisure and free time activities, family environment, PG (Lie-Bet Scale), compulsive internet use (CIUS Scale), and consumption of alcohol and cannabis were collected using a standardized questionnaire. Weighted PG prevalence was estimated in either sex, as well as the differences between various levels of PG using chi-square tests. Crude and adjusted weighted logistic regression models were used to identify predictors associated with PG. The prevalence of PG was associated with shopping frequency, compulsive internet use, cannabis use in the previous month, higher family economic status, and having a homemaker father which increased the likelihood of PG. On the contrary, cultural hobbies such as playing an instrument, painting, singing, and writing, and having a working mother were inversely associated with PG. Our results suggest that encouraging participation in creative activities along with supervised shopping and monitoring compulsive internet use and cannabis consumption may contribute some protection against adolescent PG.

**Supplementary Information:**

The online version contains supplementary material available at 10.1007/s11469-022-00950-7.

Gambling addiction has been on the rise in the European Union ever since the legalization and liberalization of gambling markets in the last decades (Calado & Griffiths, 2016). Gambling halls have proliferated in Spain and the Murcia Region, in the southern of the country. At present, Murcia Region ranks 4th with 364 gambling halls, which grew by 26% from 2016 to 2019. A meta-analysis by Storer et al. (2009) reported that problem gambling (PG) prevalence in the general population was positively associated with the density of electronic gaming machines. Games of chance are popular worldwide and are flourishing on a global scale as online gambling is growing steadily (Wood & Williams, 2011). The youth is majorly affected as young adults (over 18 years old) are more likely to play online (Gainsbury et al., 2013). The prevalence of PG in Europeans, aged 15–16 years was 1.4% according to the European School Survey Project on Alcohol and Other Drugs (ESPAD Group, 2019), and the prevalence of PG in Spanish students aged 14–18 years was 4.7% (2.0% in girls and 7.6% in boys) according to the Spanish Observatory on Drugs (OEDA. Spanish Drug Observatory, 2019). This pattern may appear in adolescents, and the younger age of onset was related to serious gambling problems (Jiménez-Murcia et al., 2010).

On the WHO website, the section on gambling (2021) notes that there are 350 million gamblers who reveal a problematic pattern each year. Additionally, it is estimated that problem gambling-related harms are threefold higher than those of drug disorder due to its impact on health, work, finance, and education (Abbott, 2017). In parallel, gambling and betting have generated a public health concern due to the increase in the number of adults seeking assistance for gambling-related problems (Suurvali, 2008). Furthermore, world population-based meta-analytic estimates have shown associations between PG and the use of alcohol, tobacco, cannabis, and illicit drugs both in younger (< 25 years old) and older adults (Dowling et al., 2017; Rash et al., 2016).

The evidence on preventive factors against gambling disorder is inconsistent. For instance, conflicting literature exists for protective and risk effects in adolescents and adults about the role of socioeconomic status and parental education and occupation concerning PG (Barnes et al., 2005; Lussier et al., 2014; Welte et al., 2008; Wood & Williams, 2011). To date, few studies have explored how free-time activities may affect gambling behavior. Behavioral addictions are closely related to leisure habits, constantly changing due to the incorporation of new technologies, which dominate the free time of adolescents and are increasingly normalized in our society. Furthermore, according to Oksanen et al. (2019), studies to understand the role of the internet and online activities on gambling behaviors are lacking. However, it has been shown that developing creative hobbies and participation in extracurricular activities can be associated with advantages for better psychological functioning and lowering addictions (smoking, alcohol, and marijuana) in youth (Guèvremont et al., 2014). Assessing the complexity of PG requires establishing methods for measuring its root causes in a broad cultural and social context. Our study will help in allocating adequate resources toward intervention strategies (Livingood et al., 2011).

Despite the gaps in the literature, addictive behavior to gambling is a multifactorial problem in adolescents with numerous risk/protective factors coming into play, such as sociodemographic determinants (being male and socioeconomic status), social problems, and substance use (poor academic performance, violence, tobacco, alcohol, cannabis, and illicit drug use), familial (parent supervision, attitudes of parents, family, and friends towards gambling), psychological (impulsivity, depression, uncontrolled temperament), or cultural factors (sedentary leisure activity, unhealthy diet pattern) (Algren et al., 2014; Dowling et al., 2017; Riley et al., 2021). We hypothesized that adolescents who socialize in a healthier environment would be less likely to develop a gambling disorder. The aim of this study was to assess the prevalence of PG and its association with family characteristics and other factors like sport, leisure-time activities, and risk-taking behaviors in adolescents.

## Materials and Methods

### Study Design, Population, and Sampling

A cross-sectional study was conducted from February to April 2019 within the ESTUDES (National Survey on Drug Use in Secondary Education Students) 2018/19 survey in the Murcia Region. The total population of this Southern Spain Region is 1.511.251 inhabitants (https://www.ine.es/). The survey has been conducted every 2 years by the Government Delegation for the National Plan on Drugs since the mid-1990s. The present fieldwork was coordinated by the National Drug Plan and Regional Drug Observatory. A two-stage cluster sampling method was employed, and a random sample of 52 public and private schools was selected from all the centers of secondary, vocational, and high schools in the Region of Murcia. In total, 104 classrooms were selected, and visits were arranged in collaboration with the school authorities. The target population was adolescents of age 14–18 years and residents of the region, totaling a student population of 58.481 in 2019. The sample was composed of 2240 pupils and had a sample error of 2.1% for a significance level of 95.5% and a population distribution at *p* = *q* = 0.5 over any substance consumption and behavioral addiction. To adapt the proportionality of the sample to the universe, the sample was weighted by the institution (whether the school was public or private) and the type of studies offered (secondary or vocational, or high school education). This survey was a part of the National Statistical Plan, and the sample of Spanish adolescents has been previously described elsewhere (Fernández-Aliseda et al., 2020).

### Instruments and Data Collection

Visits were arranged with the school authorities for each selected classroom, and, in the data collection, all students were invited to participate in a self-reported and anonymous, 45–60 min-long questionnaire. The ESTUDES questionnaire was standardized (https://pnsd.sanidad.gob.es/profesionales/sistemasInformacion/sistemaInformacion/pdf/ESTUDES_2018_Cuestionario_ALUMNOS_castellano.pdf) and found to be largely similar to that of other adolescent surveys conducted either inside Europe, such as the “ESPAD,” with Spain involved in this project, and the “Mediterranean School Survey Project on Alcohol and Other Drugs in Schools” (which is an adaptation of ESPAD in the Mediterranean region), or outside Europe, such as the US national survey “Monitoring the Future” from the National Institute on Drug Abuse for comparison purposes. The ESTUDES questionnaire was mainly designed to collect information regarding substance and non-substance use, and each scale was based on criteria for the assessment of addiction. It also included socio-demographic characteristics, parental employment, parents’ education level, academic achievement, leisure-time activities, family environment, social or health problems, perceived risk of drug use, and perceived availability of drugs, as the main variables.

### Measures and Measurement Scales

Since 2014, the ESTUDES survey has included a new module about non-substance or behavioral addictions such as gambling and addiction to new technologies, in response to the 2017–2024 National Strategy on Addictions. The prevalence of gambling for money includes engagement in at least one of the following gambling activities in the past 12 months: playing on slot machines, playing cards or dice for money, and playing the lottery or betting on sports or animal races. The question “How often (if ever) did you gamble for money in the last 12 months?” was used to compute the gambling prevalence. The existence of possible PG was screened using the validated Lie/Bet scale (Johnson et al., 1997, 1998) with satisfactory diagnostic accuracy across multiple samples (Dowling et al., 2019) and which has already been tested in the ESPAD 2015 (European adolescent survey) with a validated questionnaire (http://www.espad.org/sites/espad.org/files/5.A7_2015-Student-Master-Questionnaire_3.pdf). This scale is applicable in adolescent samples and several studies (Götestam et al., 2004; Rossow & Molde, 2006) have it demonstrated. The Lie/Bet classifies surveyed as a potential pathological gambler if the results affirmed either or both of the two DSM-IV criteria: (1) “Have you ever had to lie to people important to you about how much you gambled?” or/and (2) “Have you ever felt the need to bet more and more money?” Possible PG in a population of students aged 14–18 years, who scored 1–2 on the Lie/Bet scale. Applying a cut-off of 0–1, the responses were assigned the value of 1 for “Yes” and 0 for “No.” For other individual measures, the demographic variables and their categories were age (14–18 years), sex (male/female), school type (state or private school), mother’s employment status/father’s employment status (unemployed and others—such as pensioner, veteran, or beneficiary—, working, and homemaker), and perceived household economic situation compared to other Spanish families (upper middle, middle standard, and lower middle).

For leisure-time activities and sport variables, the following categories were used: never, a few times a year (1–3 times), at least once a month (1–3 times), at least once a week (1–7 times). The leisure items were: (1) reading books for enjoyment, (2) going out in the evening (to a disco, cafe, party…), (3) other hobbies (such as playing an instrument, singing, painting, writing…), (4) visiting adult websites (sex, violence…), and (5) shopping (online/offline). Substance consumption variables (alcohol/cannabis in the previous 30 days) were dichotomous (yes/no). And for the detection of a compulsive pattern of internet use in adolescents, the validated CIUS scale was used (compulsive internet use scale) with 14 items (Meerkerk et al., 2009) measured by a Likert scale (5 frequency categories: never, rarely, sometimes, often, very often) that consists of 56 points, and a score of at least 28 indicates the risk of compulsive internet use. These items refer to whether participants found it difficult to stop using the internet when they were online, whether they have continued using the Internet despite their intention to stop, whether they have preferred to use the Internet instead of spending time with others (e.g., parents, friends), and whether they have slept less because of using the Internet, among other questions. This questionnaire has one of the highest international reliability and is a research instrument adapted cross-culturally to the Spanish language (Lopez-Fernandez et al., 2019).

### Statistical Analyses

Descriptive statistics included numbers and prevalence of PG for the levels of selected variables, separately by sex. Prevalence was estimated as weighted percentages to adjust for differential probabilities of selection for each secondary education and vocational school strata. Differences between the variable levels were assessed using chi-squared tests. A multiple logistic regression model was applied to identify risk and protective factors of PG for all students, to obtain odds ratios (ORs) and 95% CI. A dichotomous variable was used (Lie-Bet scale: yes/no) for the crude and adjusted logistic regression model. The association between PG and parental employment status, household economic status, leisure habits, CIUS, and alcohol and cannabis consumption variables was assessed by multiple logistic regression, with PG as the dependent variable. Socio-demographic characteristics, sport, leisure context, and risky behaviors were considered as independent variables. All the statistical tests were performed using complex survey analysis and standard errors; confidence intervals and inference tests were obtained using the Taylor series linearization method and adjusted for the effects of weighting and clustering on the precision of estimates. Analyses were conducted with IBM SPSS 25.0 (IBM Corporation, Armonk, New York, USA) and Stata v14 (StataCorp, Texas, USA).

### Ethics

The study protocol complied with the Helsinki Declaration guidelines. All subjects provided informed consent, and parent consent was sought for those younger than 15 years of age, according to Spanish law.

## Results

The study sample was balanced by sex (49.2% girls, 50.8% boys). According to the Lie-Bet scale, 4.7% of subjects were affected by PG, with a significantly higher occurrence in boys (7.6%) than in girls (1.7%) (*p* < 0.001) (Table 1). The prevalence increased with age (*p* < 0.001), 3.6% and 2.6% at 14 years in boys and girls, respectively, while 19.5% and 6.8% at 18 years in boys and girls, respectively. The prevalence of PG and household economic situation, offline or online shopping, compulsive internet use, alcohol, and cannabis use in the last 30 days were statistically significantly different (*p* < 0.05) between both sexes. Also, a specific observation with boys was that reading, cultural hobbies, evening parties at discotheques or cafes, and surfing adult websites were also statistically associated with PG (Table 1).

**Table 1:** Prevalence of problem gambling and sociodemographic characteristics, leisure time and substance/non-substance use of 2240 students aged 14 to 18 years from Region of Murcia

	All	*Boys*^b^		*Girls*^b^	
		Problem gambling (Lie-Bet Scale)		Problem gambling (Lie-Bet Scale)	
*Variables*^a^	N	Yes N (%)	No N (%)	Yes N (%)	No N (%)
*Students*	2240	84 (7.6)	1050 (92.4)	18 (1.7)	1088 (98.3)
*Age (years)*					
14	458	**8 (3.6)**	**213 (96.4)**	**6 (2.6)**	**231 (97.4)**
15	621	**13 (3.7)**	**327 (96.3)**	**1 (0.4)**	**280 (99.6)**
16	594	**21 (6.0)**	**290 (94.0)**	**2 (0.6)**	**281 (99.4)**
17	454	**29 (14.3)**	**172 (85.7)**	**5 (2.3)**	**248 (97.7)**
18	113	**13 (19.5)**	**48 (80.5)**	**4 (6.8)**	**48 (93.2)**
*Mother’s employment status*					
Unemployed and others	199	12 (12.3)	95 (87.7)	3 (4.4)	89 (95.6)
Working	1402	48 (6.6)	679 (93.4)	11 (1.6)	664 (98.4)
Homemaker	588	20 (7.5)	260 (92.5)	4 (1.3)	304 (98.7)
*Father’s employment status*					
Unemployed and others	238	6 (5.3)	93 (94.7)	4 (2.6)	135 (97.4)
Working	1868	69 (7.3)	908 (92.7)	13 (1.6)	878 (98.4)
Homemaker	23	2 (29.6)	7 (70.4)	0 (0.0)	14 (100)
*Household economic situation compared to middle-income Spanish families*					
Upper middle	285	**25 (12.9)**	**168 (87.1)**	**4 (4.2)**	**88 (95.8)**
Middle standard	1844	**58 (6.7)**	**841 (93.3)**	**11 (1.2)**	**934 (98.8)**
Lower middle	90	**0 (0.0)**	**32 (100)**	**3 (6.9)**	**55 (93.1)**
*Actively participate in sports, athletics or exercising*					
Never/a few times a year (1–3 times)	314	5 (6.6)	86 (93.4)	5 (2.7)	218 (97.3)
At least once a month (1–3 times)	323	10 (7.4)	107 (92.6)	1 (0.4)	205 (99.6)
At least once a week (1–7 times)	1583	68 (7.7)	850 (92.3)	11 (1.6)	654 (98.4)
*Read books for enjoyment (no schoolbooks)*					
Never/a few times a year (1–3 times)	1281	**72 (9.4)**	**721 (90.6)**	7 (1.5)	481 (98.5)
At least once a month (1–3 times)	471	**9 (4.5)**	**185 (95.5)**	6 (2.1)	271 (97.9)
At least once a week (1–7 times)	459	**3 (2.2)**	**127 (97.8)**	4 (1.6)	325 (98.4)
*Go out in the evening (to a disco, cafe, party)*					
Never/a few times a year (1–3 times)	735	**4 (1.0)**	**359 (99.0)**	4 (1.4)	368 (98.6)
At least once a month (1–3 times)	860	**35 (8.7)**	**378 (91.3)**	8 (1.8)	439 (98.2)
At least once a week (1–7 times)	615	**43 (12.6)**	**300 (87.4)**	5 (1.7)	267 (98.3)
*Other hobbies (play an instrument, sing, draw, write)*					
Never/a few times a year (1–3 times)	1109	**55 (9.4)**	**526 (90.6)**	11 (2.3)	517 (97.7)
At least once a month (1–3 times)	351	**10 (5.9)**	**168 (94.1)**	2 (1.2)	171 (98.8)
At least once a week (1–7 times)	749	**19 (5.8)**	**338 (94.2)**	4 (1.0)	388 (99.0)
*Visiting adult websites (sex, violence)*					
Never/a few times a year (1–3 times)	1283	**15 (5.3)**	**318 (94.7)**	14 (1.6)	936 (98.4)
At least once a month (1–3 times)	305	**19 (8.1)**	**206 (91.9)**	1 (1.1)	79 (98.9)
At least once a week (1–7 times)	625	**50 (9.0)**	**510 (91.0)**	3 (4.5)	62 (95.5)
*Shopping (online/offline)*					
Never/a few times a year (1–3 times)	813	**19 (4.5)**	**426 (95.5)**	**1 (0.3)**	**374 (99.7)**
At least once a month (1–3 times)	1161	**53 (9.4)**	**519 (90.6)**	**12 (2.3)**	**582 (97.7)**
At least once a week (1–7 times)	236	**12 (11.8)**	**97 (88.2)**	**4 (2.9)**	**126 (97.1)**
*Compulsive Internet Use Scale (CIUS)*					
Yes	526	**28 (13.7)**	**183 (86.3)**	**10 (3.6)**	**305 (96.4)**
*Drinking alcohol in the last 30 days*					
Yes	1261	**65 (10.7)**	**565 (89.3)**	**16 (2.7)**	**615 (97.3)**
*Smoking cannabis in the last 30 days*					
Yes	338	**29 (15.5)**	**168 (84.5)**	**6 (4.2)**	**135 (95.8)**

^**a**^Bold values are significant (Chi-square test *p* < 0.05)

^**b**^Value presented as number (n) and prevalence (%), weighted percentage

Adjusted logistic regression analysis of socio-demographic, leisure-time activities, and substance/non-substance use factors associated with PG in the age group of 14–18 years is summarized in Table 2. In adjusted model-2 for all subjects, the likelihood of gambling for money with a problematic pattern increased with age (OR: 1.99; CI: 1.47–2.68). It was approximately 5 times more likely in boys (OR: 4.79; CI: 2.22–10.36), in families with a higher economic status (OR: 2.24; CI: 1.23–4.11), and when the father was a homemaker (OR: 5.12; CI: 1.59–16.50), while it decreased when the mother worked outside the home (OR: 0.38; CI: 0.18–0.83). The leisure factors and risk behaviors associated with a higher risk of PG were shopping (online/offline) at least once a week, 1–7 times (OR: 2.62; CI: 1.16–5.90) or once a month, 1–3 times (OR: 1.89; CI: 1.05–3.39), the compulsive internet use scale (OR: 2.95; CI: 1.64–5.33), and cannabis use in the last 30 days (OR: 1.63; CI: 1.02–2.60). The likelihood was lower in subjects practicing cultural hobbies such as playing an instrument, painting, singing, and writing at least once a week, 1–7 times (OR: 0.52; CI: 0.29–0.96) (Table 2).

**Table 2:** Adjusted logistic regressions models analyses of sociodemographic characteristics, leisure habits and substance/non-substance use associated with problem gambling of 2240 students aged 14 to 18 years from Region of Murcia

*Variables*^a^	*Problem gambling (Lie-Bet Scale)*			
	Adjusted model-1^c^ OR (CI 95%)	*p*-value	Adjusted model-2^d^ OR (CI 95%)	*p*-value
*Age*^b^				
Age (for each year of age)	**1.83 (1.41–2.37)**	** < 0.001**	**1.99 (1.47–2.68)**	** < 0.001**
*Sex*				
Girl	1 (Reference)	** < 0.001**	1 (Reference)	** < 0.001**
Boy	**5.08 (3.34–7.73)**		**4.79 (2.22–10.36)**	
*Mother’s employment status*				
Unemployed and others	1 (Ref.)		1 (Ref.)	
Working	0.49 (0.23–1.02)	0.057	**0.38 (0.18–0.83)**	**0.016**
Homemaker	0.50 (0.24–1.04)	0.063	0.55 (0.26–1.14)	0.107
*Father’s employment status*				
Unemployed and others	1 (Ref.)		1 (Ref.)	
Working	1.29 (0.55–3.03)	0.553	1.64 (0.58–4.66)	0.344
Homemaker	3.29 (0.59–18.36)	0.171	**5.12 (1.59–16.50)**	**0.007**
*Household economic situation compared to middle-income Spanish families*				
Middle standard	1 (Ref.)		1 (Ref.)	
Upper middle	**2.60 (1.57–4.30)**	** < 0.001**	**2.24 (1.23–4.11)**	**0.010**
Lower middle	1.25 (0.36–4.33)	0.719	1.46 (0.43–5.01)	0.538
*Actively participate in sports, athletics or exercising*				
Never / A few times a year (1–3 times)	1 (Ref.)		1 (Ref.)	
At least once a month (1–3 times)	0.63 (0.30–1.33)	0.218	0.43 (0.18–1.04)	*0.061*
At least once a week (1–7 times)	1.11 (0.59–2.09)	0.741	0.97. (0.51–1.85)	0.928
*Read books for enjoyment (no schoolbooks)*				
Never / A few times a year (1–3 times)	1 (Ref.)		1 (Ref.)	
At least once a month (1–3 times)	0.66 (0.37–1.16)	0.147	0.92 (0.50–1.70)	0.746
At least once a week (1–7 times)	0.46 (0.19–1.11)	*0.081*	0.63 (0.21–1.88)	0.401
*Go out in the evening (to a disco, cafe, party)*				
Never / A few times a year (1–3 times)	1 (Ref.)		1 (Ref.)	
At least once a month (1–3 times)	**3.58 (1.41–9.12)**	**0.009**	2.07 (0.71–6.06)	0.179
At least once a week (1–7 times)	**5.41 (2.33–12.58)**	** < 0.001**	2.62 (0.90–7.66)	*0.077*
*Other hobbies (play an instrument, sing, paint, write)*				
Never / A few times a year (1–3 times)	1 (Ref.)		1 (Ref.)	
At least once a month (1–3 times)	0.60 (0.29–1.24)	0.163	0.53 (0.22–1.27)	0.152
At least once a week (1–7 times)	**0.57 (0.34–0.95)**	**0.032**	**0.52 (0.29–0.96)**	**0.036**
*Shopping (online/offline)*				
Never / A few times a year (1–3 times)	1 (Ref.)		1 (Ref.)	
At least once a month (1–3 times)	**2.67 (1.63–4.37)**	** < 0.001**	**1.89 (1.05–3.39)**	**0.033**
At least once a week (1–7 times)	**3.47 (1.79–6.74)**	** < 0.001**	**2.62 (1.16–5.90)**	**0.021**
*Visiting adult websites (sex, violence)*				
Never / A few times a year (1–3 times)	1 (Ref.)		1 (Ref.)	
At least once a month (1–3 times)	1.40 (0.76–2.57)	0.272	0.86 (0.40–1.82)	0.682
At least once a week (1–7 times)	1.78 (0.84–3.79)	0.129	0.97 (0.46–2.03)	0.932
*Compulsive Internet Use Scale (CIUS)*				
No	1 (Ref.)		1 (Ref.)	
Yes	**2.56 (1.55–4.21)**	** < 0.001**	**2.95 (1.64–5.33)**	** < 0.001**
*Drinking alcohol in the last 30 days*				
No	1 (Ref.)		1 (Ref.)	
Yes	**2.84 (1.69–4.77)**	** < 0.001**	1.72 (0.71–4.15)	0.222
*Smoking cannabis in the last 30 days*				
No	1 (Ref.)		1 (Ref.)	
Yes	**2.57 (1.56–4.22)**	** < 0.001**	**1.63 (1.02–2.60)**	**0.040**

^**a**^Bold values are significant (Chi-square test p<0.05)

^**b**^OR per one-year increase of age

^**c**^Adjusted model-1: adjusted OR by sex, age, school type, and selected variable

^**d**^Adjusted model-2: adjusted OR by sex, age, school type, parental occupation, household economic situation, sport, cultural and electronic leisure, compulsive internet use scale, alcohol, and cannabis use

## Discussion

The prevalence of PG among adolescents (in the last 12 months) was higher among boys than girls (7.6% versus 1.7%) in the age group of 14 to 18 years (*p* < 0.001). The observations from the current study concur with the findings from previous reports about a higher prevalence in boys (7.6%) versus girls (2.0%) in Spanish adolescents within this age range (OEDA. Spanish Drug Observatory, 2019) as well as in almost all the European countries (6.3% for boys vs. 2.4% for girls on average) among from 15- to 16-year-olds who had gambled in the last 12 months (ESPAD Group, 2019). Furthermore, the prevalence of PG is in line with another US survey, being significantly higher in boys (3.3%) than in girls (0.9%) (Welte et al., 2008). Unsurprisingly, the risk of PG increases with age and is higher in males (the odds are 5 times higher); these results are consistent with prior research (Calado et al., 2017; Ekholm et al., 2014).

With respect to family characteristics, a higher economic status perceived of the household was a risk factor for adolescent PG. Contradictory evidence exists about this association. While a study found that adolescent problem gamblers are more likely to come from the lower social class (Griffiths & Wood, 2000), in another longitudinal study from the USA, Barnes et al. (2005)  found that lower socioeconomic status (SES) was weakly associated with higher levels of gambling only in females. Such outcomes could be due to the greater accessibility of gambling in neighborhoods with lower socioeconomic conditions. A higher prevalence of gambling opportunities, as well as neighborhood environmental risk, was associated with PG (Lussier et al., 2014; Pearce et al., 2008). Nevertheless, previous studies (Buja et al., 2019; Welte et al., 2008) found a dose–response association between having money (e.g., higher weekly income) and gambling among adolescents, and the study led by Welte et al. (2008) did not establish a relationship between PG and low SES. Besides, Turner et al. (2011) noted that subjects who were involved in heavy gambling were the ones who could spend the money freely or without any economic constraints. Considering these researchers, our results could be explained by the hypothesis that teenagers with higher economic status perception are likely to spend money on gambling because they perceive having enough money to spend.

Meanwhile, the hypothesis for the reason behind a working mother as a protective factor might be that these mothers being informed about adolescent risky behaviors monitor their children carefully. Although, conversely, the mother who works outside the home may not protect her children of risky behaviors, such as adolescents’ smoking, alcohol intake, and cannabis use, due to reduced supervision at home (Buja et al., 2019; Moñino-García et al., 2018). Parental monitoring may reduce the risk of adolescent gambling (Castrén et al., 2021) and may lead to long-term effects on a decreased likelihood of substance use (Kristjansson et al., 2010). The risk factor concerned with the father working as a homemaker should be considered as the possible effect of the economic status of the family as the father is unemployed. A study by Livazović and Bojčić (2019) did not establish parent employment as a significant predictor of adolescent PG, while a mother’s employment status was only found to be associated with satisfaction in family life. This study with multivariate models has shown that parent employment status could be significant for increasing or decreasing gambling risk in adolescents. As the literature is scarce, we highlight the need for additional research exploring the influence of the family environment on gambling patterns in adolescents. Thus, the family role should be further investigated (McComb & Sabiston, 2010).

According to the multivariate model, the likelihood of PG increases with cannabis use in the previous month. This result is consistent, given that the association between cannabis use and pathological gambling in adolescents is well established in the literature (Canale et al., 2016). Similar conclusions were drawn from a metanalysis by Dowling et al. (2017). Cannabis use was identified as a longitudinal risk factor for the development of gambling problems among adolescents, as well as alcohol use frequency. Nonetheless, contrary to our predictions, alcohol use was not independently related to PG. However, considerable evidence links PG to adolescent alcohol use (Buja et al., 2019; Riley et al., 2021).

To our knowledge, after controlling all the other potential predictors, this is the first study showing shopping (online or offline) and compulsive internet use are risk factors for PG in adolescents, and practicing creative activities is protective, such as playing an instrument, painting, singing, and writing (1–7 times per week). Adolescents with a compulsive pattern of the internet have a threefold higher vulnerability to PG. The new technologies play a crucial role in facilitating the development of PG in youth. Olason et al. (2011) highlight that a higher proportion of problem gamblers is found among internet gamblers in contrast to non-internet gamblers. And in an era where the youth have easier access to a range of gambling influences (Welte et al., 2008), the change from an offline gambling pattern to an online one is foreseeable (Abbott, 2017) due to its accessibility, affordability, and anonymity (Calado et al., 2017). Oksanen et al. (2019) revealed that competent gambling (online gambling, card games in general, and betting on games involving personal skill, such as billiards) was associated with compulsive internet use and further stated that the new forms of online gambling are potential risks for younger generations. Estevez et al. (2015) found significant differences between groups with and without pathological gambling with elevated scores for compulsive shopping among gamblers. This result is also supported by Kausch (2003), but the association was among adult gamblers. Conversely, no differences were found between internet addiction and gambling (Estevez et al., 2015).

This study was not without limitations. One of the most important limitations is its cross-sectional design, which means that causality among the variables cannot be assured. On the other hand, we have a small sample of girls with problem gambling (*n* = 18) for this study, so it is difficult to obtain a statistically significant contrast. The adjusted analysis model was for both sexes, but there were differences between boys and girls, as well as the effect of the sex variable and interaction effects (see figures in Appendix A) . Although anonymity to participants was guaranteed, social desirability bias might affect self-reporting and hence data accuracy. The study has the advantage of being a large probability sample from our region, which allows comparison with other results, although differences in the study designs for PG complicate making comparisons across studies. Also, the sample design was adapted to the universe (the target population). In addition, part of the variables studied is based on the ESPAD questionnaire that has been validated, especially for substance use questions and behavioral addiction scales, as anticipated. The ESTUDES questionnaire has not been cross-culturally adapted in the Spanish language. Finally, another advantage is establishing a pre-COVID-19 baseline for future studies on gambling in adolescent samples.

Various studies conducted in Iceland have shown that accessible and structured leisure-time opportunities contributed to an extraordinary decrease in substance use and abuse among adolescents, similar to our results. Long-term preventive interventions in family, school, peers, and a healthy leisure environment, such as parental monitoring of evening and night entertainment, increased adolescent involvement in free time activities, participation in sports supervised by adults, and encouraging children and adolescents to spend time with their families also helped in better well-being (Kristjansson et al., 2016, 2020). However, no association was observed between the practice of sports and PG in the present study. In the words of Frieden (2010), “changing the context to make individual’s default choices healthy” facilitates implementing long-term primary prevention of adolescent substance use. In a Canadian cross-sectional study (14–17 years), participation in non-sport activities (including clubs or groups in music, art, drama, photography, Scouts, or student council activities) was associated with positive academic outcomes and with a reduced likelihood of having tried alcohol, smoking, and marijuana (Guèvremont et al., 2014). It is known that alcohol or substance use and gambling share analogous characteristics, and their social consequences are quite similar (Leeman & Potenza, 2012). Canale et al. (2016) proposed that theoretical models for substance use might also apply to gamble. Likewise, as hypothesized, our results suggest that leisure habits may play a key role in reducing the probability of developing a possible problem of gambling in adolescents, which is an age in which peer pressure is high and maladaptive behavioral patterns can be molded (Monaghan & Wood, 2010). Therefore, facilitating a healthier environment could have health and social benefits in preventing gambling-related harms and future risk-taking behaviors.

As mentioned earlier, our results provide useful information for the prevention of gambling addiction, and they represent an opportunity to examine the influence of modern life leisure activities, including information and communication technologies, on problem gambling, and their repercussions in adolescents. Indeed, our results provide for the first time evidence of an association between adolescent problem gambling and several leisure time factors. They also point out the important role of parents in monitoring and paying attention to potential compulsive patterns of internet use and shopping (1–7 times per week) of their adolescent children. Further research is warranted to confirm the potential influence of leisure-time behaviors on attitudes towards gambling in teenagers in prospective study settings.

In conclusion, prevention and health promotion programs geared toward cultural hobbies, monitoring shopping, compulsive internet use, and cannabis consumption may lead to a decrease in the levels of PG in teenagers, especially in boys. Regulatory and preventive strategies are needed which should focus on environmental actions as well as individual behavioral changes at an early age. Consequently, based on our findings, we recommend regional authorities and stakeholders responsible of health promotion policies to invest in cultural activities (e.g., music activities, or story-writing and short film competitions) as potential interventions to avoid or reduce the engagement of adolescents in gambling activities, to which they are prone when they exhibit unhealthy behaviors, such as compulsive internet use or cannabis consumption. Future studies of these factors would be useful for deepening the comprehension of the problems of gambling among adolescents. Additional research is also required for gauging the causes of interregional differences in the risks associated with PG (Huang & Boyer, 2007).

## Supplementary Information

Below is the link to the electronic supplementary material.

Supplementary file1 (DOCX 154 KB)
