# Supplementary material for: The Adolescent Problem Gambling Prevalence Associated with Leisure-Time Activities and Risky Behaviors in Southern Spain
Source: Int J Ment Health Addict. 2022 Nov 18:1–15. Online ahead of print. doi: 10.1007/s11469-022-00950-7 (PMC9676736; doi:10.1007/s11469-022-00950-7)
Supplement: Supplementary file 1 — Supplementary file1 (DOCX 154 KB) [file 11469_2022_950_MOESM1_ESM.docx]

**Appendix A. Supplementary figures**

Supplementary figure 1. Prevalence of problem gambling and sex, age group and household economic situation^a^ in a representative sample of 2240 students from the Region of Murcia.


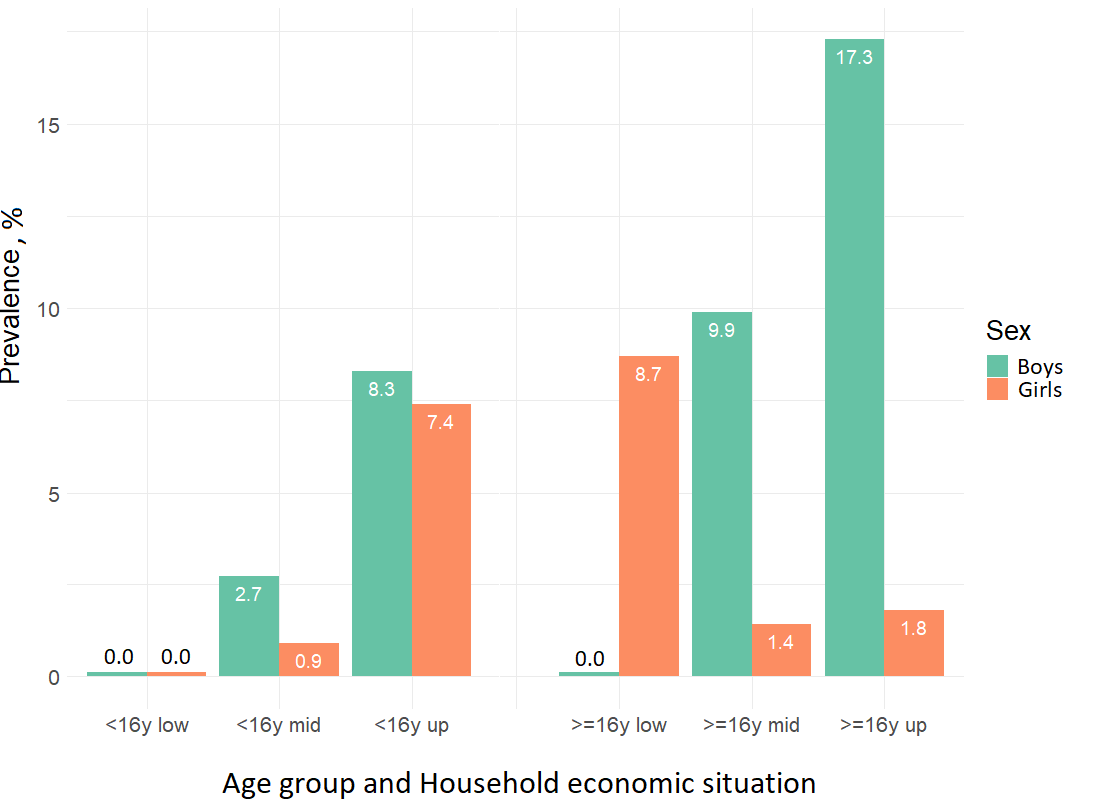


ª^The combination between age group (from 14 to 15 years old,<16y, and over 16 years old, ≥16y) and household economic situation (lower middle, middle standard and upper middle).^

Supplementary figure 2. Prevalence, with 95% confidence intervals^a^, of problem gambling and sociodemographic characteristics, leisure-time and substance/non-substance use, by sex, in a representative sample of 2240 students from the Region of Murcia.


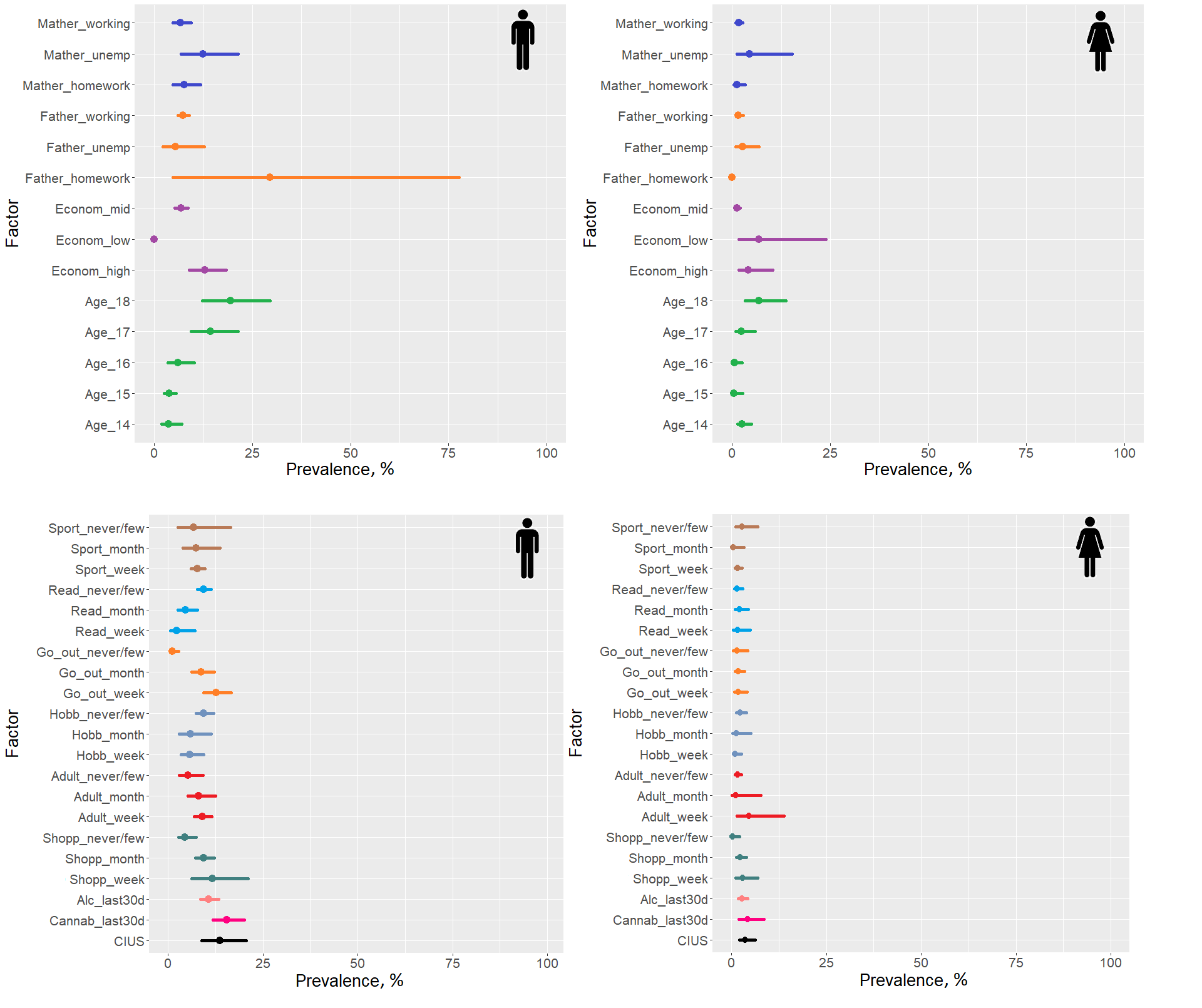


ª^The factor variables are represented according to colour change in each sex.^
